# Leukocyte Proliferation and Immune Modulator Production in Patients with Chronic Kidney Disease

**DOI:** 10.1371/journal.pone.0073141

**Published:** 2013-08-09

**Authors:** Ladan Mansouri, Josefin M. Paulsson, Ali Moshfegh, Stefan H. Jacobson, Joachim Lundahl

**Affiliations:** 1 Unit of Clinical Immunology and Allergy, Department of Medicine, Karolinska University Hospital Solna, Karolinska Institutet, Stockholm, Sweden; 2 Department of Oncology-Pathology, Karolinska University Hospital Solna, Karolinska Institutet, Stockholm, Sweden; 3 Department of Clinical Sciences, Danderyd Hospital, Karolinska Institutet, Stockholm, Sweden; University of Florida, United States of America

## Abstract

**Introduction:**

In Chronic Kidney Disease (CKD), immune cells are affected by uremic retention toxins. Given this effect, we analyzed lymphocyte proliferative response and immune modulators production following *in vitro* stimulation.

**Methods:**

Whole blood was drawn from healthy controls, patients with eGFR <20 ml/min/1.73 m^2^ (Pre-dialysis, CKD stages 4 and 5) and hemodialysis patients (stage 5D). Peripheral cells were incubated for six days with pokeweed mitogen, concanavalin A, Staphylococcus enterotoxin A or influenza A vaccine. Peripheral lymphocyte proliferation was then analyzed by the “Flow-cytometric Assay of Specific Cell-mediated Immune response in Activated whole blood” (FASCIA) method, and cytokine profile in the cell supernatants was analyzed by the Milliplex multi-array method.

**Results:**

The absolute number of lymphoblasts in response to mitogenic stimulation and the number of cells in each CD4+ and CD8+ subpopulation were similar comparing the three groups, except for a single decline in number of lymphoblasts after stimulation with Staphylococcus enterotoxin A, comparing dialysis patients with healthy controls. Levels of interleukin (IL)-2 (p=0.026), -10 (p=0.019) and -15 (p=0.027) in the Staphylococcus enterotoxin A-stimulated supernatant were lower in hemodialysis patients compared to healthy controls. Levels of IL-15 (p=0.017) from pre-dialysis patients and levels of IL-5 (p=0.019) from hemodialysis patients in influenza A vaccine-stimulated supernatants were also lower compared to controls. In pokeweed mitogen–stimulated supernatant, IL-2 levels (p=0.013) were lower in hemodialysis patients compared to pre-dialysis patients. TNF-α, IL-10, IL-12, IL-15, IL-8, MCP-1, IP-10, IFN-α2, IL-1α and eotaxin levels were all significantly higher in plasma obtained from CKD patients.

**Conclusion:**

Our results suggest that T-cells from CKD patients have similar proliferative response to stimulation compared with healthy individuals. Moreover, however the immune cells show inability to produce selected cytokines, most likely due to the uremic milieu or dialysis procedure.

## Introduction

The main causes of morbidity and mortality in patients with chronic kidney disease (CKD) are related to cardiovascular diseases, infections and other conditions originating in improper regulation of the immune system [1,2]. In CKD patients, both the innate and the adaptive immune systems are affected due to retention of uremic toxins and therapeutic interventions [3,4]. A number of studies have shown disturbances in the function of polymorphonuclear cells (PMNs), monocytes, macrophages and dendritic cells (DCs) as well as in T-lymphocytes and B-lymphocytes [5]. Affected processes involve cellular activation and proliferation, phagocytosis, cytokine production, antigen-presentation, suppressive regulation and apoptosis [6–8]. These disturbances are also reflected in the peripheral level of immune modulators, which is characterized by a pro-inflammatory profile [9].

As regards the T-lymphocytes in CKD, there is a variety of findings on the net number of circulatory lymphocytes, the lymphoproliferative response to stimulators and cytokine production [10–12]. In most of studies, a conventional proliferation assay has been applied, using purified peripheral blood mononuclear cells (PBMCs) primarily [10,11,13]. This method includes purification steps that might impact the in vitro response [14,15]. In addition, the concept of a pro-inflammatory milieu is often based on analysis of selected mediators only [4,10].

Based on the pivotal role of CKD on T-lymphocyte dysfunction and the lack of consistent data in the literature, we applied a method called “Flow-cytometric Assay of Specific Cell-mediated Immune response in Activated whole blood” (FASCIA). This method uses whole blood and flow-cytometric analysis of lymphoblast proliferation [16–18]. A major advantage is that the cells have been less manipulated and pre-activated ex vivo. We used this method to measure the proliferative response to selected stimulators in cells from CKD patients. Moreover, we applied a multi-array method to analyze the cytokine profile in peripheral blood and following stimulation. Patients with CKD stages 4 and 5 (Pre-dialysis), patients on maintenance hemodialysis (CKD stage 5D) and healthy individuals were enrolled. Our results on immune dysregulation in CKD patients warrants further studies to enhance the understanding of the causative mechanisms of CKD-related outcomes, such as increased susceptibility to infections and increased risk of cardiovascular complications.

## Materials and Methods

### 1: Study population

The patients were recruited from the Department of Nephrology at the Karolinska University Hospital in Stockholm, Sweden. The patients had the diagnosis of Chronic Kidney Disease with an estimated Glomerular Filtration Rate (eGFR) of <20 ml/min/1.73 m^2^ (Pre-dialysis Group, CKD stages 4 and 5) (n=14) or were undergoing hemodialysis, three times/week for four to four and a half hours/dialysis procedure using polysulfone high-flux dialyzer (Dialysis Group, CKD 5D) (n=14). The duration of dialysis in this group was 2 to 17 years with median duration of 5 years, and the residual GFR ranged from 4 to 12 and median of 8.5 ml/min/1.73 m^2^. GFR was estimated according to the MDRD formula [19]. Dialysis was done through Arteriovenous fistula (n=7), Graft (n=3) or Central dialysis catheter (n=4) accesses. The purity of the dialysate fluid was tested at recommended intervals according to the European best practice guidelines at the hospital [20] and was found to be pure at all occasions. Patients with diabetes, cancer, ongoing infection or chronic inflammatory disease (such as rheumatological diseases) and those taking immunosuppressive drugs were excluded. Healthy subjects were recruited among healthy blood donors (n=14) and were age-matched with patients. Demographic characteristics of participants are shown in Table **1**. The causes of CKD in pre-dialysis patients were; Adult dominant-polycystic kidney disease (AD-PKD) (n=6), medullary cystic kidney disease (n=2), focal segmental glomerulosclerosis (FSGS) (n=1), renal vascular disease due to hypertension (n=2), IgA nephropathy (n=2), Chronic tubulointerstitial nephritis (n=1) and the causes in hemodialysis patients were; Adult dominant-polycystic kidney disease (AD-PKD) (n=1), renal vascular disease due to hypertension (n=1), Glomerulonephritis (n=1) and CKD of unknown etiology (n=11). All the pre-dialysis and half of the dialysis patients were treated for high blood pressure with a diuretic agent, B2 blocker agent, angiotensin-converting-enzyme inhibitor (ACE inh.), angiotensin receptor blocker (ARB) or a combination of these drugs (Table **1**). Participants filled out a questionnaire regarding their health status, current co-morbidities, medication and vaccination status against Hepatitis and Influenza virus. Written informed consent was obtained from all participants.

**Table 1:** Demographic characteristics of participants.

Category	Subcategory	Healthy subjects	Pre dialysis	Hemodialysis
Sex	Male (n)	7	9	7
	Female (n)	7	5	7
Age range/median (years)		33-65/54	33-65/51	36-66/53.5
Co-morbiditis (n)	Asthma/ Allergy	-	2	4
	Epilepsy	-	-	1
	Parkinson’s disease	-	-	1
	History of heart disease	-	1	3
	High blood pressure	-	14	7
FH^*^ of kidney disease(n)		-	8	4
Treatment (n)	ACE inh^**^/ ARB^***^		13	4
	ESA^****^>		6	12
	Intravenous Iron		1	9
	Oral Iron		4	0
	Vitamin D		11	10
Smoking (n)		-	0	3
Swedish snuff (n)		-	3	2

^*^ Family History

^**^ Angiotensin Converting Enzyme inhibitor

^***^ Angiotensin Receptor Blockers

^****^ Erythropoiesis Stimulating Agents

### 2: FASCIA

The FASCIA method was employed for the detection of T-lymphocyte proliferation and lymphoblast formation [16]. Whole blood was drawn (before dialysis procedure in hemodialysis patients) into sodium Heparin tubes (Vacutainer, Becton Dickinson, UK) and diluted 1:9 in RPMI 1640 medium (Gibco, UK) with the addition of 2 mM L-glutamine (Gibco, UK), 10 000 IU/ml Penicillin (Gibco, UK) and 10 000 µg/ml Streptomycin (Gibco, UK). Blood was also taken for the collection of plasma to be stored at -80° C in aliquots for further analysis. The blood-medium suspension was distributed in polystyrene round bottom tubes with caps (Falcon, Becton Dickinson, NJ, USA) and the cells were subjected to stimulation. One tube was incubated with cell culture medium alone and is referred to as the control. The following stimuli were used in the final concentration: Pokeweed mitogen (PWM, Sigma-Aldrich, Germany) 1 µg/ml, Concanavalin A (Con A, Sigma-Aldrich, Sweden) 1µg/ml, Staphylococcus enterotoxin A (SEA, Sigma-Aldrich, Germany) 100 ng/ml and Influenza A vaccine (IAV, Fluarix, GlaxoSmithKline AB, Sweden) diluted 1:100. Prepared stimuli (200 µl) were added to blood-medium suspensions (1800 µl) and 200 µl of the medium was added to the control tube. The tubes were incubated for six days in a cell culture incubator at 37 °C with 5% CO2 and loose caps. After the incubation period, 1400 µl of the supernatant was collected for cytokine analysis. The cells were resuspended in the remaining medium and the cell suspension was distributed into three tubes (100 µl per tube); one tube for staining with monoclonal antibodies (isotype controls IgG1 and IgG2b), one tube with anti CD3-PC5, CD4-RD1, CD8-ECD and CD45-FITC (CYTO-STAT, tetra CHROME, Beckman Coulter Inc., USA) and one tube for cell counting.

Antibodies (10 µl from each) were added and the tubes were incubated for 15 min in darkness at room temperature. The erythrocytes were then lysed by the addition of 2 ml isotonic solution (154 mM NH_4_Cl, 10 mM KHCO_3_ supplemented with 0.1 mM EDTA, pH 7.2). After 5 min the tubes were centrifuged for 5 min at 300g in 4 °C, the supernatant was discarded and the cells were washed with Phosphate buffered saline (PBS). Following additional centrifugation, the supernatant was removed and cells were suspended in 350 µl of cold PBS and subsequently analyzed. Cell count was performed by the use of cell counting beads (Flow-Count Fluorospheres, Beckman Coulter Inc., USA). 100 µl of beads were mixed with 100 µl of the cells prior to analysis. Acquisition was conducted by a flow-cytometer (Navios, Beckman Coulter Inc., Hialeah, FL, USA) and the data was analyzed by the Kaluza Analysis Software (Beckman Coulter Inc., USA).

**Figure 1:**
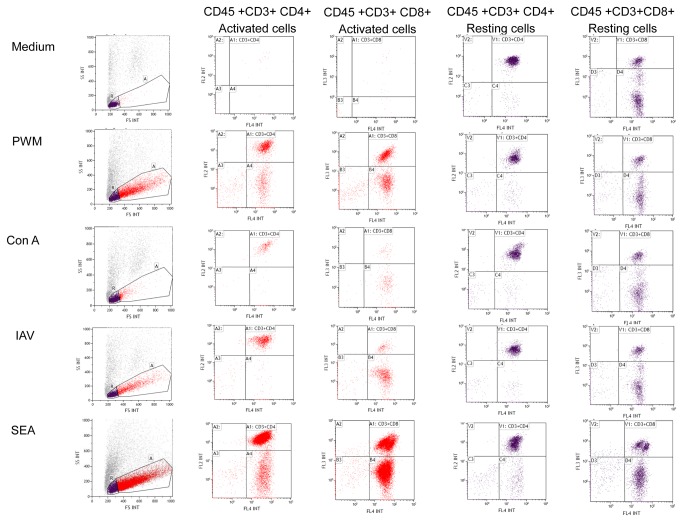
Flow-cytometric analysis of cell-mediated immune response in activated whole blood. The FITC-CD45+ resting and activated leukocytes from medium, Pokeweed mitogen-, Concanavalin A-, Influenza A vaccine- and Staphylococcus enterotoxin A-stimulated supernatants were gated according to their size and granularity on forward and side scatter plot (in region R and A respectively). The CD3+ CD4+ cells and CD3+ CD8+ cells were detected within the regions R and A accordingly.

The resting and activated leukocytes were gated according to their size and granularity on forward, side scatter and FITC-CD45+ staining, as shown in Figure **1**. The gates were set based on the medium control with defined resting cells in region R and activated blasts in region A. CD3+CD4+ and CD3+CD8+ cells were detected within the regions R and A according to the gates set by the respective isotype controls. The percentage of the cells in each area was calculated by flow-cytometer. Calculation of the absolute number of resting and activated leukocytes was performed using flow-count beads. The absolute numbers of different subpopulations (CD3+CD4+ and CD3+CD8+ cells) were subsequently calculated based on the relative distribution and percentage.

### 3: Characterization of soluble immune modulators in plasma and culture supernatants

The concentrations of immune modulators in culture supernatants and plasma were detected by Milliplex 26-plex (Millipore Corp, St. Charles, Missouri, USA) according to the manufacturer’s protocol. Eotaxin, Granulocyte colony-stimulating factor (G-CSF), Granulocyte-macrophage colony-stimulating factor (GM-CSF), Interferons (IFN)-α2, IFN-γ, Interleukin (IL)-10, IL-12 (p40), IL-12 (p70), IL-13, IL-15, IL-17, IL-1α, IL-1β, IL-2, IL-3, IL-4, IL-5, IL-6, IL-7, IL-8, Interferon gamma-induced protein (IP)-10, monocyte chemotactic protein (MCP)-1, Macrophage inflammatory protein (MIP)-1α, MIP-1β, Tumor necrosis factor (TNF)-α and TNF-β were assessed. This method was applied to a broad range of immune modulators in order to reveal different aspects of the immune response to lymphocyte stimulation. The concentration of cytokines in the supernatants of stimulated cells was normalized against the medium control.

### 4: Statistical analysis

Scatter plots were prepared by GraphPad Prism 5, representing 25-75% interquartile range, a line at the median and whiskers at the non-outlier values. Statistical analysis was done in GraphPad Prism 5 and STATISTICA version 10 (Stat Soft, Inc., USA). Since values were not normally distributed in all groups, comparison between the three groups in terms of laboratory data, number of activated lymphoblasts, subpopulations and immune modulator levels was performed by Kruskal-Wallis test. Significant differences between groups were analyzed using the post hoc Multiple Comparisons p values (2-tailed) test. A p<0.05 was considered significant.

### 5: Ethics Statement

The study was approved by the local Ethical Committee at the Karolinska University Hospital, Stockholm, Sweden (Dnr 2011/113-31/3). Written informed consent was obtained from all participants.

## Results

### 1: Laboratory findings

The laboratory data of the study participants are shown in Table **2**. There were no significant differences between the three groups with regard to total leukocyte and platelet counts. Erythrocyte count was significantly higher in healthy controls compared with patients. No significant differences were observed between the three groups in terms of C-reactive protein (CRP); however, serum albumin was significantly lower in hemodialysis patients compared to pre-dialysis patients and healthy subjects.

**Table 2:** Laboratory data for blood parameters.

Category	Healthy subjects^*^	Pre dialysis^*^	Hemodialysis^*^
Leukocyte count (×10^9^/L)	5.9 (4.8-6.7)	5.7 (4.7-7.5)	7 (5.7-10.3)
Erythrocyte count (×10^12^/L)	4.8 (4.6-5)	4.2^a^ (3.7-4.4)	4.1^b^ (3.6-4.5)
Platelet count (×10^9^/L)	255 (202-286.5)	192.5 (146.5-260.8)	228 (135.8-276.8)
Creatinine (µmol/L)	67 (58.5-81.5)	438.5^c^ (329-683.5)	586^d^ (401-906.3)
Albumin (g/L)	39 (38-43)	38.5 (35.7-40.5)	34^e^,^f^ (32-35.5)
C-reactive protein (mg/L)	1.8 (0.4-2.3)	2.5 (2-4.2)	2.9 (1.4-6.3)

^*^ n=14

^a^ p=0.003 compared to healthy subjects

^b^ p=0.01 compared to healthy subjects

^c^ p=0.0002 compared to healthy subjects

^d^ p<0.0001 compared to healthy subjects

^e^ p=0.0004 compared to healthy subjects

^f^ p=0.045 compared to pre-dialysis patients

Values are given as median and interquartile range (25-75%). Kruskal-Wallis test was used for comparison between the three groups. Significant differences between the groups were analyzed using the post hoc Multiple Comparisons p values (2-tailed) test. p<0.05 was considered significant.

### 2: Detection of lymphoblasts in whole blood by the FASCIA method

A difference (p=0.041) was observed in the absolute number of lymphoblasts after stimulation with SEA, comparing hemodialysis patients with healthy controls but there were no significant differences in the absolute number of lymphoblasts between the healthy controls, pre-dialysis patients and hemodialysis patients after stimulation with any one of the following stimulators: ConA, IAV and PWM (Table **3**). The absolute number and percentage of the CD4+ and CD8+ cells in the activated population were calculated and no significant differences were observed between the three groups (Tables **4**, **5**).

**Table 3:** Absolute number of activated lymphoblasts (cells/µl).

Category	SEA	ConA	IAV	PWM
Healthy subjects	27015 (21649-41925)	2086 (1264-3437)	3803 (1900-5824)	13286 (6929-18522)
Pre dialysis	19909 (7080-36776)	2503 (1763-3568)	2180 (1212-4203)	16124 (7219-21602)
Hemodialysis	15593 (9989-20346)	3846 (1417-6092)	2226 (1146-3957)	8393 (5341-10145)
Significance	p=0.041^a^	ns*	ns	ns

^a^ ns: non significant

^*^ Comparing healthy controls and hemodialysis patients

The absolute number of lymphoblasts (cells/µl) was calculated after stimulation with SEA, ConA, IAV and PWM for six days. Values are given as median and interquartile range (25-75%). Kruskal-Wallis test was used for comparison between the three groups. Significant differences between groups were analyzed using the post hoc Multiple Comparisons p values (2-tailed) test. p<0.05 was considered significant.

**Table 4:** Absolute number and percentage of CD4+ cells in the activated lymphoblasts (cells/µl).

Category	SEA	ConA	IAV	PWM
Healthy subjects	18311 (14880-30002)	1423 (981-2805)	2033 (976-3201)	5301 (3274-10163)
	69 (5-85)%	73 (4-86)%	68 (2-89)%	48 (9-75)%
Pre dialysis	13819 (6620-29523)	2108 (1516-2547)	1815 (769-2858)	7030 (3347-14297)
	73 (40-83)%	83 (43-89)%	78 (10-96)%	55 (42-76)%
Hemodialysis	10035 (7176-15777)	2583 (969-4510)	1411 (905-3214)	4467 (2118-5987)
	70 (61-89)%	68 (46-93)%	78 (27-96)%	59 (10-84)%
Significance	ns*	Ns	ns	ns

^*^ ns: non significant

The absolute number and percentage of CD4+ cells in the activated populations (cells/µl) were calculated after stimulation with SEA, ConA, IAV and PWM for six days. Values are given as median and interquartile range (25-75%). Kruskal-Wallis test was used for comparison between the three groups. Significant differences between groups were analyzed using the post hoc Multiple Comparisons p values (2-tailed) test. p<0.05 was considered significant.

**Table 5:** Absolute number and percentage of CD8+ cells in the activated lymphoblasts (cells/µl).

Category	SEA	ConA	IAV	PWM
Healthy subjects	8110 (6423-9539)	327 (133-696)	400 (168-804)	2472 (1044-3411)
	29 (1-40)%	16 (1-34)%	13 (0-23)%	17 (0-39)%
Pre dialysis	7335 (3177-10954)	296 (124-442)	136 (83-306)	2047 (1289-4381)
	27 (14-58)%	9 (3-38)%	6 (1-25)%	20 (10-43)%
Hemodialysis	3762 (2161-6150)	722 (191-1229)	200 (106-394)	1801 (959-2520)
	26 (9-43)%	20 (3-46)%	11 (2-37)%	22 (4-54)%
Significance	ns*	Ns	ns	ns

^*^ ns: non significant

The absolute number and percentage of CD8+ cells in the activated populations (cells/µl) were calculated after stimulation with SEA, ConA, IAV and PWM for six days. Values are given as median and interquartile range (25-75%). Kruskal-Wallis test was used for comparison between the three groups. Significant differences between groups were analyzed using the post hoc Multiple Comparisons p values (2-tailed) test. p<0.05 was considered significant.

There were also no significant differences between the three groups when the absolute number of lymphoblasts was normalized against leukocyte count in peripheral blood.

### 3: Detection of soluble molecules and immune modulators in the plasma and culture supernatants

**Figure 2:**
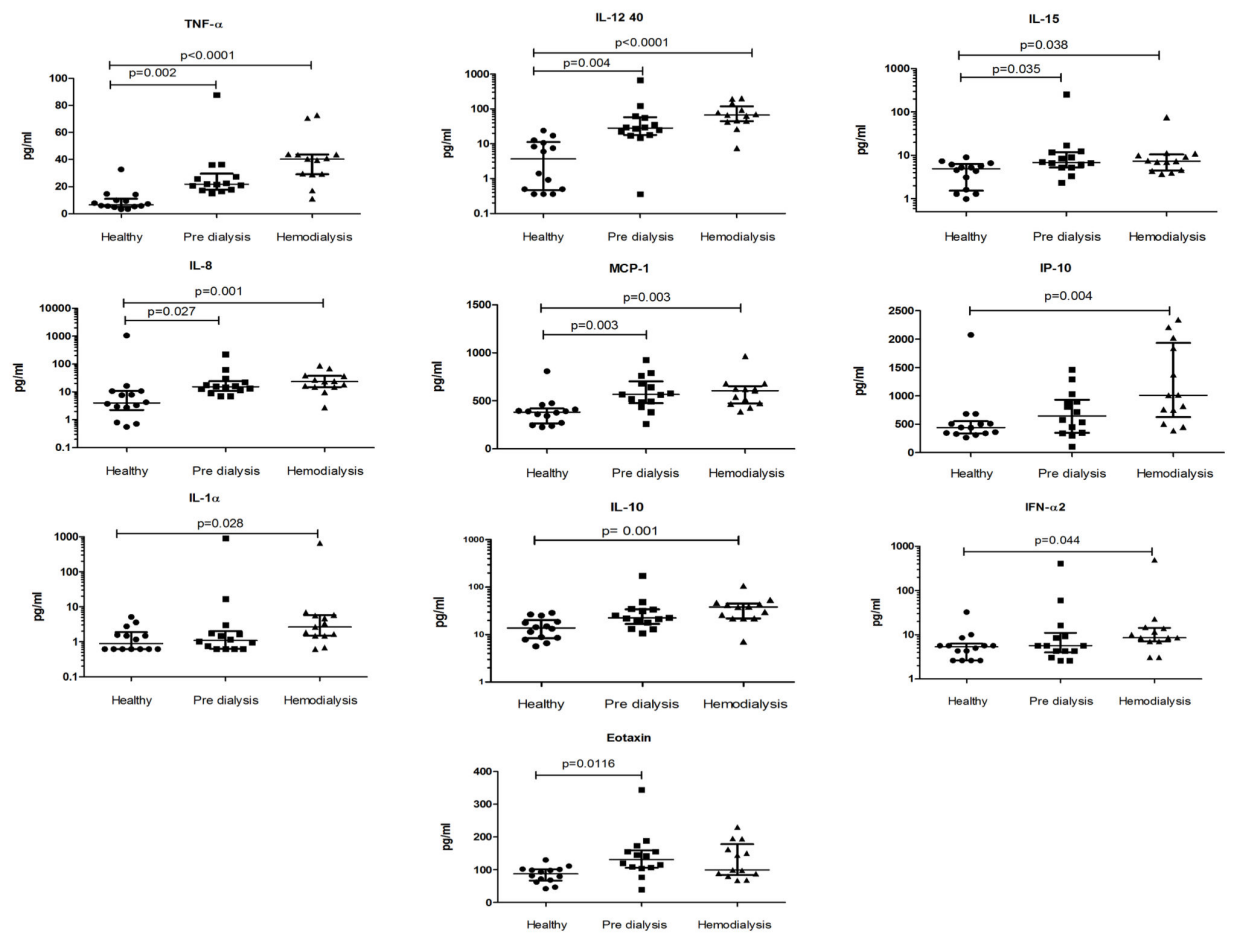
Scatter plots showing cytokines with significantly different plasma concentrations (pg/ml). The concentration of soluble immune modulators in the plasma of healthy controls, pre-dialysis patients and hemodialysis patients were analyzed and compared. Whiskers represent 25-75% interquartile range with the median shown by a line. Kruskal-Wallis test was used for comparison between the three groups. Significant differences between groups were analyzed using the post hoc Multiple Comparisons p values (2-tailed) test. p<0.05 was considered significant.

**Figure 3:**
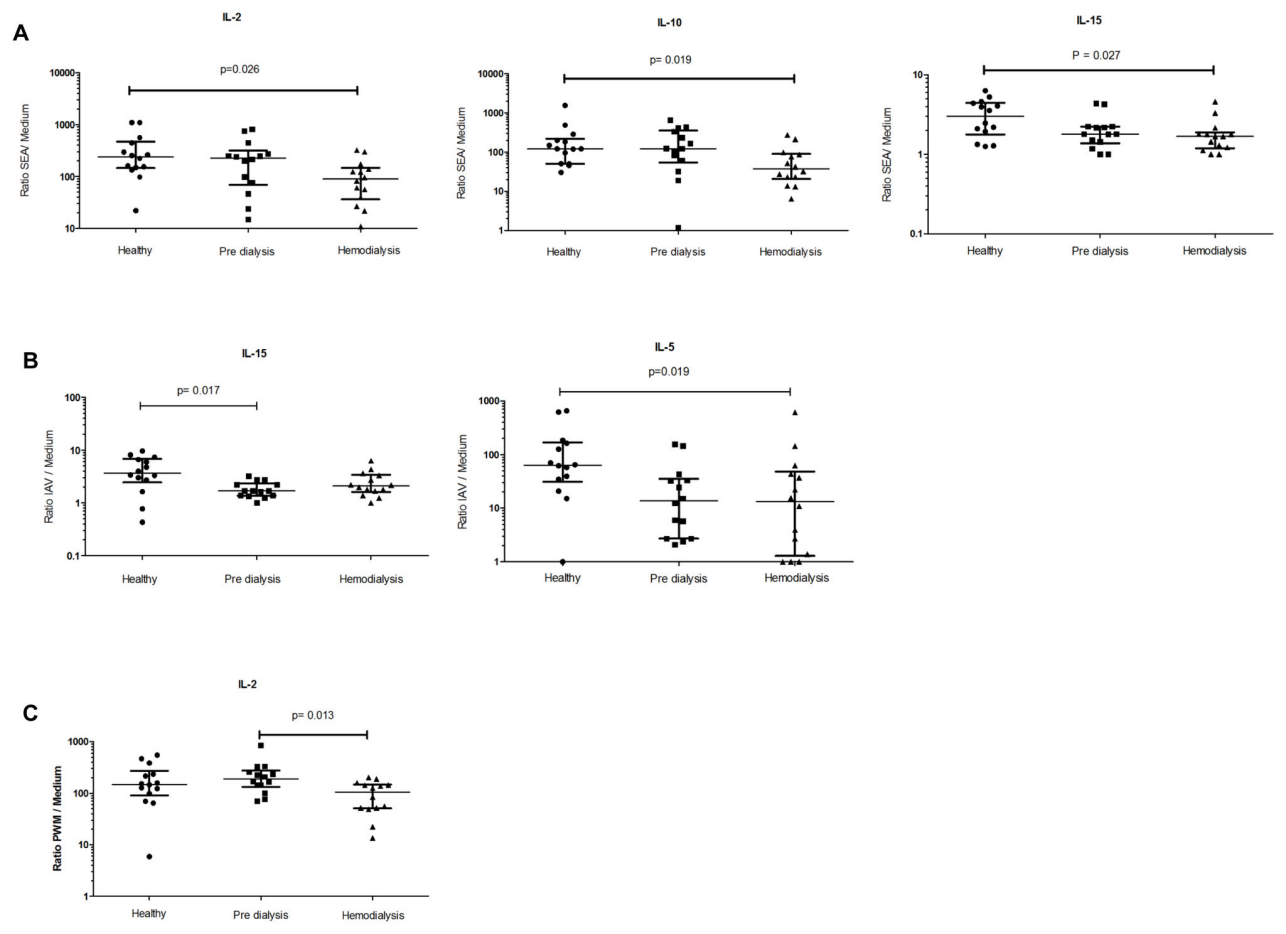
Scatter plots showing significantly different ratios (immune modulator concentration in supernatants to medium). The concentration of immune modulators in all the cultured supernatants (SEA, ConA, IAV, PWM and medium) was analyzed and the ratio of cytokine concentration in each supernatant to medium was calculated. The three groups of healthy controls, pre-dialysis patients and hemodialysis patients were compared. The results from cultured cells with SEA (A), IAV (B) and PWM (C) are shown respectively. Whiskers represent 25-75% interquartile range with the median shown by a line. Kruskal-Wallis test was used for comparison between the three groups. Significant differences between groups were analyzed using the post hoc Multiple Comparisons p values (2-tailed) test. p<0.05 was considered significant.

The plasma levels of Eotaxin, IFN- α2, IL-1 α, IL-8, IL-10, IL-12 p40, IL-15, IP-10, MCP-1 and TNF-α differed significantly between the three groups (Figure **2**). The ratios of immune modulators in the supernatants to medium control were calculated (Figure **3**). IL-2, IL-10 and IL-15 ratios were significantly lower in supernatant from SEA-stimulated cells in hemodialysis patients compared with cells from healthy controls. In the supernatant from cultured cells with IAV, the IL-5 ratio was significantly lower in hemodialysis patients while the IL-15 ratio was significantly lower in pre-dialysis patients comparing to healthy subjects. The IL-2 ratio was also found to be significantly lower in the supernatant from cultured cells with PWM in hemodialysis patients comparing to pre-dialysis patients.

## Discussion

The aim of the present study was to address potential immune dysregulations in terms of T-cell proliferation and cytokine profile in CKD patients. We report a similar total number of circulatory leukocytes, absolute number of lymphoblasts and CD4+ and CD8+ subpopulations in response to stimulators comparing healthy controls with pre-dialysis and hemodialysis patients, except for a single decrease in number of lymphoblasts in hemodialysis group after stimulation with SEA compared with healthy controls. We also report significantly lower levels of IL-2, IL-10 and IL-15 in the SEA co-culture supernatant from hemodialysis patients compared to healthy controls, as well as lower levels of IL-15 from pre-dialysis patients and lower levels of IL-5 from hemodialysis patients in the IAV co-culture supernatants. The level of IL-2 in the PWM co-culture supernatant of hemodialysis patients was lower compared to pre-dialysis patients. But moreover, the analysis of immune modulators in plasma showed significantly higher concentrations of TNF-α, IL-10, IL-12 40, IL-15, IL-8, MCP-1, IP-10, IFN-α2, IL-1α and Eotaxin in CKD patients.

In the present study, we assessed T-lymphocyte proliferative response, using the FASCIA method. This method has been used to assess lymphoproliferation in response to different infectious microorganisms or following vaccination [18,21–23]; however, this is the first time this method has been used for assessment of immune dysregulation in CKD. FASCIA is a convenient method, since only a small amount of whole blood is needed and the results are reproducible. An advantage is that the cells are not manipulated by the isolation process and the cell culture composition resembles the in vivo condition [14–16]. Studies that have compared whole blood assays with PBMC assays also state that whole blood assays can be a good alternative [24,25]. However, some studies revealed that the PBMC isolation procedure such as Ficoll-Hypaque separation could alter the functional characteristics of T-lymphocytes [14], which in turn may lead to less responsiveness following stimulation but despite this, several studies have used PBMC in proliferation assays [10,22].

We report no significant differences in the absolute number of lymphoblasts and T-lymphocyte subsets between the three groups after stimulation. Data on the proliferation capacity in CKD patients varies in the current literature, depending on the stimuli which have been used and cell subtypes been analyzed. In a study by Lisowska et al. [13], the authors revealed a diminished percentage of CD4+ CD28+ (a major co-stimulatory molecule for IL-2 production) proliferating cells after stimulation with anti-CD3 and Stachowski et al. [26] found a blunted proliferation of uremic PBMCs after stimulation with anti-CD3. Moreover, Ankersmit et al. [10] revealed a reduced T-lymphocyte proliferative response to stimulators in hemodialysis patients compared with healthy subjects. However, in line with present data, van Riemsdijk et al. [11,12] demonstrated that the proliferation capacity of lymphocytes in hemodialysis patients was the same as in healthy controls. Thus, there are incongruent findings regarding T-lymphocyte activity and proliferation in patients with CKD. We should also consider that there is a complex of signaling pathways and due to different settings and use of various mitogens, different pathways are engaged which leads to diverse cell responses. Besides different experimental set-ups, differences in the patient selection can also impact the results and interpretation of data. In our study, the treatment with erythropoiesis stimulating agents (ESA) in 12 out of 14 hemodialysis patients may affect the results, since it has been suggested that treatment with these agents can improve the phenotype and proliferation property in CD4+ T cells [27]. In addition, treatment with ACE inh. /ARBs in patients should be considered since studies have revealed effect of these drugs on modulation of Th1, Th17-cell function [28] as well as TNF-α, IL-6, MCP-1 serum levels [29].

We analyzed soluble inflammatory molecules in the supernatants and plasma using Milliplex 26-plex. This method has the advantage of assessing a large number of immune modulators simultaneously, which enabled us to observe different aspects of the immune response. Some cytokines were detected at lower concentrations in the co-culture supernatants of pre-dialysis and hemodialysis patients compared to those from healthy controls. It is known that the expansion of antigen-specific T-lymphocytes necessitates proliferation and release of cytokines such as IL-2, IL-15, and IL-7 [30]. IL-2, IL-10 and IL-15 are, in turn, involved in B-lymphocyte proliferation and differentiation [31,32]. Interestingly, we found decreased levels of IL-2, IL-10 and IL-15 in the supernatant of SEA stimulated co-cultures. A lower production of these cytokines may lead to disruption of the proliferation and antibody production of B-lymphocytes, followed by a lower response to vaccination and higher vulnerability to infections in CKD patients. One limitation of the whole blood method is that all the cells are present in co-cultures and therefore the cytokine production cannot be referred to a specific cell type.

High levels of TNF- α, IL-12, IL-15, IL-8, IP-10, IFN-α2 and IL-1α in plasma of CKD patients indicate activation and increased production of cytokines by blood cells such as monocytes, macrophages and dendritic cells. This leads to an inflammatory state as previously discussed [33–35], and could be linked to the fact that atherosclerosis and cardiovascular diseases are the most common causes of morbidity and mortality in CKD patients [1]. Higher levels of TNF-α, IL-12, IL-15, IL-8 and MCP-1 in both pre-dialysis and hemodialysis groups may illustrate that uremic milieu can lead to an increase in the level of these cytokines, independently of the dialysis procedure. Higher levels of IP-10, IFN-α2 and IL-1α in hemodialysis patients may be a consequence of the interaction between blood and the dialyzer membrane [9]. On the other hand, a higher level of IL-10 reveals an ongoing immunoregulatory process, the purpose of which is to oppose the effect of inflammatory cytokines [36]. The high plasma levels of this cytokine in patients (but low levels in culture supernatants) could be suggestive of exhaustion in terms of IL-10 production and an aberration in the pro anti-inflammatory adjustment. We also observed higher levels of Eotaxin in pre-dialysis patients comparing with healthy individuals. This finding might refer to engagement of other cell subsets such as eosinophils and basophils in the pathogenesis of CKD outcomes. However, this observation needs to be further investigated. It has been suggested that uremia, dialysis procedures and infections in CKD patients can lead to activation and a stress-induced premature senescence (SIPS) in a number of mononuclear cells and these cells, in turn, release a large amount of cytokines. Thus, SIPS mononuclear cells are possibly both the cause and the result of the chronic inflammatory state in this patient group [37].

By combining the FASCIA and Milliplex methods, we investigated the proliferation and cytokine production of T-lymphocytes in response to stimulators in patients with CKD. The T-cell proliferation was equal. However, the cells had a reduced capacity to produce immune modulators in response to mitogens. This may indicate exhaustion or inability to produce cytokines due to the uremic milieu, or it may be a consequence of therapeutic intervention (e.g. dialysis). We also report a systemic status of inflammation in CKD patients with increased levels of pro-inflammatory cytokines. These immune dysregulations warrant further investigations to enable a better understanding of the causative mechanisms of disease-related consequences such as increased susceptibility to infections, low response to vaccinations and higher risk of cardiovascular disease.
